# INPRESS: INvasive PRESSure Monitoring Knowledge and Practice Survey

**DOI:** 10.1111/nicc.70443

**Published:** 2026-03-12

**Authors:** Oliver Coolens, Rolf Dubb, Arnold Kaltwasser, Ulf Lorenzen, Peter Nydahl

**Affiliations:** ^1^ Academy of the District Hospitals Reutlingen gGmbH Reutlingen Germany; ^2^ Department of Anesthesiology and Intensive Care Medicine University Hospital Schleswig‐Holstein Kiel Germany; ^3^ Nursing Research, University Hospital of Schleswig‐Holstein Kiel Germany; ^4^ Institute of Nursing Science and Practice, Paracelsus Medical University Salzburg Austria

**Keywords:** haemodynamic monitoring, monitoring, safety, transducer, vital sign

## Abstract

**Background:**

In patients on Intensive Care Units (ICU), invasive arterial pressure monitoring with closed transducer systems is widely used, yet the accuracy of measurement depends on correct zeroing, levelling and interpretation of arterial waveforms.

**Aim:**

The aim was to assess the knowledge and practice of clinicians (nurses, physicians, anaesthesia/operation technicians) working in critical care, operating theatres and emergency departments about arterial blood pressure assessment and managing the closed transducer system.

**Study Design:**

A cross‐sectional online survey was conducted in Germany in May 2025. The questionnaire included 26 single‐ and multiple‐response items on zeroing, transducer level, arterial waveform, phlebostatic axis and practical handling of the arterial line system. Data were analysed using descriptive statistics and Fisher's exact test to assess associations between knowledge and professional experience or institutional setting.

**Results:**

A total of 303 clinicians participated, the majority being ICU nurses *n* = 245 (81%), with *n* = 21 (7%) answering all questions correctly. The percentage of correct answers in multiple questions were in the sections about zeroing procedures *n* = 34–48 (11%–16%), transducer reference levels *n* = 5–232 (2%–77%), arterial pressure waveform *n* = 33–53 (11%–17%) and phlebostatic axis identification *n* = 11–101 (4%–33%). Recognition of hydrostatic, gravitational effects was higher *n* = 232 (77%), but scenario‐based questions were correctly answered by only half. Practical handling showed variation, with *n* = 147 (48%) reporting correct pressure bag inflation and *n* = 147 (48%) avoiding heparin in flush solutions. More years of experience and employment in tertiary centres were associated with higher accuracy.

**Conclusions:**

Clinicians demonstrated limited knowledge of invasive blood pressure monitoring, with significant variability across domains. Structured education, protocols and simulation‐based training are needed to improve accuracy and safety.

**Relevance to Clinical Practice:**

Strengthening haemodynamic monitoring competence is essential to ensure reliable, homogenous measurements, reduce errors and enhance patient safety.

## Impact Statements

- What is known about the topic
  ○ Accurate haemodynamic monitoring is essential for the evaluation of therapy and ensuring patient safety.
  ○ Knowledge deficits in zeroing, levelling and waveform interpretation have been reported, but systematic assessment across professions is limited.
- What this paper adds
  ○ In a survey among clinicians, knowledge gaps in invasive arterial blood pressure monitoring, especially regarding transducer reference levels and the phlebostatic axis, were present.
  ○ Professional experience and employment in tertiary centres are associated with higher accuracy in haemodynamic assessment.
  ○ Structured education, protocols and simulation‐based training are needed to ensure patients' safety and reliable monitoring practice.

## 1 Background

Patients in intensive care units (ICU) are critically ill and frequently haemodynamically unstable, requiring close monitoring and timely interventions. Continuous assessment of vital parameters, including heart rate, blood pressure, breathing frequency and oxygen saturation, is essential to guide clinical decision‐making. Haemodynamic monitoring plays a central role in this process, as it provides information about circulatory status and organ perfusion. Blood pressure measurement—reported as systolic, diastolic and mean arterial pressure—remains one of the most widely used indicators. Haemodynamic monitoring refers to the measurement of functional characteristics of the heart and circulatory system that influence tissue perfusion [1]. In practice, blood pressure can be assessed using non‐invasive methods (palpation, cuff‐based oscillometry) (with high risk of error), most commonly by invasive catheterisation via the radial artery with a closed transducer system, or more recently by waveform analysis using finger sensors [2]. Accurate assessment and interpretation of these values are fundamental for optimising therapy, preventing complications and ensuring the safety of patients in intensive care settings [3, 4].

Even when invasive arterial pressure monitoring provides continuous data, documentation in many clinical environments—whether manual or Patient Data Management System (PDMS)‐supported—remains intermittent, which may limit recognition of rapid haemodynamic changes [5].

Training in haemodynamic assessment in critical care is vital, as accurate interpretation of parameters such as blood pressure directly informs critical treatment decisions, including the titration of vasoactive drugs and initiation of fluid therapy [6]. The use of closed transducer systems requires knowledge and training. Clinical competence in this area is influenced by multiple factors: the level of experience, the presence of structured educational programmes, adherence to institutional protocols and access to advanced monitoring technologies [1, 7]. Enhancing training ensures timely recognition of circulatory instability, supports safe therapeutic interventions and ultimately improves patient outcomes and safety [1].

Clinicians in ICU, operating theatres and emergency departments receive educational training how to manage haemodynamic assessment of critical patients and to apply the pressure transducer in correct positions. Assessments of this knowledge are infrequent, and when conducted, they consistently reveal only moderate proficiency. Hence, we aimed to survey the knowledge and self‐reported practice of clinicians working in critical care, operating theatres and emergency departments about arterial blood pressure assessment and managing the closed transducer system. Inherent anatomical and physiological variability in arterial blood pressure across measurement sites [8, 9, 10] must be distinguished from avoidable procedural errors such as incorrect zeroing or levelling, which represent the primary focus of this study.

## 2 Methods

A cross‐sectional study design with an online survey using a convenience sample was chosen. The survey was performed in Spring 2025 in Germany among clinicians (nurses, physicians, anaesthesia/operation technicians) working in critical care, operating theatres and emergency departments and distributed via the authors' personal and professional networks. Since the survey was anonymous, voluntary and addressed to clinicians only, ethical approval was not necessary. The study was performed following the declaration of Helsinki. The survey was designed and reported in concordance with the CROSS checklist [11].

### 2.1 Questionnaire

The questionnaire was based on the publication by Schortgen and Le Bec (2025) [12]. The questionnaire comprised in total 26 questions, 19 with single answer options and 7 with multiple answers options. In addition to collecting sociodemographic characteristics (e.g., institution size, workplace, professional group, years of experience and prior involvement in research or teaching related to arterial pressure monitoring), the survey included questions on the measurement procedure (purpose and rationale of zeroing, transducer level), the morphology of the arterial pressure waveform, the phlebostatic axis and the practical management of the arterial line system (e.g., use of heparinised flush solutions, pressure bag settings). In total, the questionnaire comprised 6 categories: participant characteristics, zeroing procedure, transducer level, arterial pressure waveform, phlebostatic axis and practical handling of the arterial line system (Supplement). Correct answers were not disclosed to participants.

### 2.2 Translation and Pretest

The questionnaire developed by Schortgen and Le Bec [12] was translated into German and subsequently back‐translated to ensure content equivalence [13]. The questionnaire was adapted to the national context of the participants and to reflect an interprofessional treatment approach. These adaptations were evaluated for content validity by an expert panel [14]. A face validity assessment was conducted with seven experts, and minor adjustments were made to the questionnaire's wording [15].

### 2.3 Study Population and Setting

The survey was addressed to ICU clinicians. Included were all clinicians (physicians, nurses, anaesthesia/operation technicians), working in ICU, operation theatres or Emergency Departments. In the country of the survey, most patients on ICU are equipped with arterial lines in the radial artery to ensure a continuous blood pressure management and access to blood samples. The required knowledge and management are communicated during the study and specialisation for critical care, and practically during the first days of ICU training, and are also presented in common medical books (e.g., Larsen et al. [16]). In general, nurses and physicians are responsible for the management of arterial blood pressure management, but not therapists, students or interns.

Respondents were provided with detailed information regarding the estimated time required to complete the survey. Participation was voluntary and anonymous, and no personal data except years of professional experience were collected.

### 2.4 Survey Distribution

The survey was distributed via emails and posts in professional and social network of the authors from 15 April 2025, to 15 May 2025. Due to this method of distribution, a calculation of a response rate was not feasible. The call for participation included the title, aim, content, target group, a link to the survey and the authors' names. The survey's call was distributed once with a reminder after two weeks. There were no incentives offered.

We used SurveyMonkey (https://de.surveymonkey.com/r/INPRESS2025) for the survey. To ensure participation from multiple clinicians from single ICUs and hospitals, multiple responses via a similar IP‐address was feasible. IP‐addresses were not stored. In the survey, participants could click for‐ and backwards until they finished the survey. There was no information about correct or wrong answers. Today, the survey in German is still open as quiz‐mode and shows at the end the correct answers to participants and can be used free of charge by anyone.

### 2.5 Statistical Analysis

The anonymised data collected via the web‐based version of the online survey tool SurveyMonkey were exported to IBM SPSS Statistics (Version 29.0.2.0 [17]) for statistical processing. Nominal and continuous variables were presented as absolute and relative frequencies.

The questionnaire's items were designed either as multiple‐response sets, allowing participants to select more than one answer with multiple correct options, or as single‐response formats, offering several answer choices including ‘I do not know’, with only one correct option. Non‐responses were treated as incorrect.

For improved comparability, two clusters were created. Professional experience was categorised as ‘≤ 5 years’ and ‘> 5 years’, based on the assumption that longer experience generally correlates with greater expertise [18]. In addition, institution sizes were grouped into ‘tertiary and quaternary care’ versus ‘primary and secondary care’, reflecting the assumption that larger facilities are likely to perform a higher number of arterial pressure measurements.

Significance testing for categorical variables was performed using Fisher's exact test only. A significance level of *α* = 0.05 was applied [19, 20]. Given that the majority of respondents were nurses and only very few participants represented other professional groups, meaningful subgroup stratification by profession was not possible.

For all multiple‐response items, we additionally calculated partial credit and penalised partial credit scores to quantify the proportion of correctly selected options, with and without correction for incorrect choices. Furthermore, item difficulty (*p*‐values) was calculated for all dichotomously scored items as the proportion of respondents who selected the correct answer, with higher *p*‐values indicating easier items.

## 3 Results

A total of 303 individuals participated in the survey and could be evaluated. Among the participants, *n* = 210 (70%) worked in ICU and *n* = 63 (21%) in anaesthesiology. The majority *n* = 243 (81%) were nursing professionals, of whom *n* = 219 (73%) reported more than five years of professional experience. Up to five years of experience had *n* = 81 (27%) participants (Table 1).

**TABLE 1:** Socio‐demographical data.

Item	*n* (%)
In which setting do you work?	
Other	14 (5)
Intermediate care	11 (4)
Anaesthesia	63 (21)
Intensive care unit	210 (69)
Emergency department	3 (1)
Missing	2 (1)
Which professional group do you belong to?	
Other	17 (6)
Physician	2 (1)
Nurse	243 (80)
Advanced practice Nurse	11 (4)
Anaesthesia/operation technician	28 (9)
Missing	2 (1)
How many years of work experience do you have (excluding training)?	
< 6 months	23 (8)
6 months–2 years	16 (5)
> 2–5 years	42 (14)
> 5 years	219 (72)
Missing	3 (1)
In what type of institution do you work?	
Other	6 (2)
Primary care hospital	16 (5)
Secondary care hospital	46 (15)
Tertiary care hospital	56 (19)
Quaternary (maximum care) hospital	177 (58)
Missing	2 (1)
Have you ever been involved in designing research studies on hemodynamics?	
Yes	4 (1)
No	293 (97)
I don't know	3 (1)
Missing	3 (1)
Have you ever taught a course on hemodynamics in the intensive care unit?	
Yes	35 (12)
No	266 (88)
Missing	2 (1)

*Note:* Characteristics of respondents. Frequencies. *n* = 303.

### 3.1 Correct Answers

In total, *n* = 21 (7%) answered all questions correctly. Participants with more than 5 years of experience answered *n* = 16 (7%) of all questions correctly, vs. *n* = 5 (6%) of participants with less than 5 years of experience. The overall correct answers are presented in Table 2.

**TABLE 2:** Correct answers—overall.

Item		Answer	
		Correct	Incorrect
		*n* (%)	*n* (%)
Zeroing	What is the purpose of zeroing?^a^	34 (11)	269 (89)
	Zeroing must be performed …^a^	48 (16)	255 (84)
Transducer level	The height above the floor at which the transducer is mounted^a^	5 (2)	298 (98)
	If the transducer is placed too far above the zero reference level. …	233 (77)	70 (23)
	If the transducer is placed too far below the zero reference level …	232 (77)	71 (23)
	Where do you place the transducer connected to the radial arterial catheter?	63 (21)	240 (79)
	In which figure(s) is the transducer (shown in white) placed at the correct reference level?^a^	23 (8)	280 (92)
	If the transducer is at position 1. What MAP is displayed?	162 (53)	141 (57)
	If the transducer is at position 2. What MAP is displayed?	47 (15)	256 (85)
Arterial pressure waveform	What is your assessment and what actions would you take?^a^	33 (11)	270 (89)
	The invasive blood pressure measurements presented on the monitor reflect …	74 (24)	229 (76)
	Using a fluid‐filled system prevents a water column effect on blood pressure.	53 (17)	250 (83)
Phlebostatic axis	The phlebostatic axis …^a^	11 (4)	292 (96)
	Which anatomical landmark identifies the phlebostatic axis in a supine patient?	101 (33)	202 (77)
	Validity of Phlebostatic Axis in Different Positions^a^	59 (19)	244 (81)
	To measure cerebral perfusion pressure (MAP – ICP), place the arterial transducer …	67 (22)	236 (78)
	If the transducer is placed at the level of the right atrium. the displayed arterial pressures …	109 (36)	194 (64)
Practical handling	To what pressure do you inflate the pressure bag in your practice (mmHg)?	147 (48)	156 (52)

^a^ Multiple responses. Frequencies. Missing responses were considered incorrect. Correct answers were predefined. Each question had multiple answers, correct answers can be found in the supplement. *n* = 303.

### 3.2 Zeroing Procedure

A total of *n* = 34 (11%) of participants correctly answered the question regarding the purpose of zeroing (multiple responses), and *n* = 48 (16%) correctly identified the requirement for zeroing (multiple responses).

### 3.3 Transducer Level

The question concerning the transducer mounting height above the floor (multiple responses) was answered correctly by *n* = 5 (2%). When asked how the displayed blood pressure would change with an adjustment of the transducer height above the reference level, *n* = 233 (77%) responded correctly. Respondents with more than 5 years of experience did this significantly more often correctly (94% vs. 76%, *p* < 0.001).

When the transducer was placed below the reference level, *n* = 232 (77%) correctly identified the corresponding change in the displayed blood pressure. 63 (21%) of participants were able to indicate the correct height for transducer placement when connected to an arterial catheter. 23 (8%) of participants correctly answered the question regarding the reference level for correct transducer positioning (multiple responses).

The question, ‘The current mean arterial pressure (MAP) is 60 mmHg. If the transducer is at position 1, what MAP is displayed in mmHg?’ was answered correctly by *n* = 162 (53%). Participants with longer professional experience (61% vs. 73%, *p* = 0.007) and those working in central or maximum care facilities (35% vs. 74%, *p* = 0.007) were more likely to respond to this question correctly.

When the transducer was positioned at position 2 with a given MAP of 60 mmHg, *n* = 47 (15%) of participants correctly identified the displayed blood pressure. In this item the proportion of correct responses was likewise higher among respondents from larger hospitals (25% vs. 6%, *p* = 0.005).

### 3.4 Arterial Pressure Waveform

The correct measures that could be derived from an arterial pressure waveform were identified by *n* = 33 (11%) of participants. A total of *n* = 53 (17%) correctly evaluated the statement that a fluid‐filled arterial measurement system prevents a hydrostatic column effect on the transducer and that the hydrostatic column effect represents the pressure of a fluid column on the transducer in a closed system as false. Participants from larger institutions were significantly more likely to provide the correct answer to this question (11% vs. 28%, *p* = 0.019).

### 3.5 Phlebostatic Axis

In total, *n* = 11 (4%) correctly answered the question regarding the phlebostatic axis and its function. The anatomical landmark for identifying the phlebostatic axis in the supine position was known to *n* = 101 (33%) of respondents. Participants with greater professional experience identified the phlebostatic axis significantly more often (30% vs. 54%, *p* = 0.002).

In the present study, *n* = 59 (19%) of respondents correctly named the supine position as the valid anatomical reference for determining the phlebostatic axis. Experienced professionals provided this response significantly more frequently (9% vs. 23%, *p* = 0.003).

Correct placement of the transducer at the level of the tragus for cerebral perfusion pressure measurement was reported by *n* = 67 (22%). The statement ‘If the transducer is placed at the level of the right atrium, the displayed arterial pressures approximately reflect the pressure at the aortic root’ was classified as correct by *n* = 109 (36%).

### 3.6 Practical Handling

In *n* = 147 (48%) of cases, the pressure bag of the arterial system was inflated to 300 mmHg. The transducer was most commonly mounted on a dedicated plate, as reported by *n* = 165 (55%) (Table 3). A total of *n* = 188 (65%) of respondents stated that they never add heparin to the flushing solution in the system's pressure bag, and *n* = 8 (3%) always added heparin (Table 4).

**TABLE 3:** Transducer.

Where do you most commonly place the transducer in your practice?	
Item	*n* (%)
On the patient's arm	26 (8)
On a mounting plate	165 (55)
On the patient's body near the arterial catheter insertion site	8 (3)
On the thorax	5 (2)
I don't know	4 (1)
Missing	95 (31)

*Note:* Transducer placement in practice. Frequencies. *n* = 303.

**TABLE 4:** Heparin.

Do you add heparin to the pressure bag of the arterial system in your setting?	
Item	*n* (%)
Always	8 (3)
Sometimes	7 (2)
Never	188 (62)
I don't know	6 (2)
Missing	94 (31)

*Note:* Heparin in the flush bag. Frequencies. *n* = 303.

### 3.7 Partial Credit Score

Item difficulty was calculated using both partial credit (pc) and penalised partial credit (pc_pen) scoring. Partial credit represents the proportion of correct response options selected, whereas the penalised variant additionally accounts for incorrectly selected options.

Items q7, q13, q16 and q21 showed moderate difficulty (pc ≈0.40–0.60), while q8 was comparatively easy (pc = 0.82) but exhibited a substantially lower penalised score (pc_pen = 0.16), indicating frequent co‐selection of distractors. Item q19 demonstrated consistently low difficulty across both scoring methods (pc = 0.15; pc_pen = 0.14), reflecting either a conceptually demanding task or suboptimal distractor performance. Taken together, the partial credit analyses provide additional psychometric insight into the functioning of the instrument and help identify items that may benefit from further refinement (Table 5).

**TABLE 5:** Partial credit scores.

	*N*	Min	Max	Mean	SD
Partial credit scores for multiple‐response items					
pc Q2	303	0.00	1.00	0.6238	0.28189
pc Q3	303	0.00	1.00	0.8185	0.38608
pc Q4	303	0.00	1.00	0.3927	0.25663
pc Q8	303	0.00	1.00	0.4389	0.29813
pc Q10	303	0.00	1.00	0.5083	0.36573
pc Q13	303	0.00	1.00	0.1529	0.27878
pc Q15	303	0.00	1.00	0.4323	0.49622
Penalised partial credit scores for multiple‐response items					
pc_pen Q2	303	0.00	1.00	0.5182	0.27184
pc_pen Q3	303	0.00	1.00	0.1584	0.36573
pc_pen Q4	303	0.00	1.00	0.3630	0.26096
pc_pen Q8	303	0.00	1.00	0.4026	0.31042
pc_pen Q10	303	0.00	1.00	0.3806	0.35102
pc_pen Q13	303	0.00	1.00	0.1408	0.26985
pc_pen Q15	303	0.00	1.00	0.1947	0.39664

*Note:* Partial credit scores were calculated as the number of correct response options selected divided by the total number of correct options for each item. Penalised partial credit scores were calculated as (correct options − incorrect options)/maximum correct options, truncated at 0 to avoid negative values. Higher values reflect better item performance.

### 3.8 Item Difficulty

Item difficulty (*p*‐values) was calculated as the mean proportion of correct responses for each dichotomously scored item. The *p*‐values ranged from very low (e.g., *p* = 0.02 for Q4) to high (e.g., *p* ≈0.89 for Q5 and Q6), indicating substantial variability in item difficulty across the instrument. Several items showed low *p*‐values (e.g., Q2, Q3, Q8, Q13), reflecting high difficulty, whereas others yielded moderate to high *p*‐values (e.g., Q9/1, Q14, Q17, Q19), representing easier items. This range of item difficulty is typical for knowledge‐based assessments and suggests that the instrument captures both basic and more complex aspects of invasive pressure monitoring (Table 6).

**TABLE 6:** Item difficulty.

Item difficulty (*p*‐values) for all dichotomously scored items					
	*N*	Min	Max	Mean	SD
Q2	303	0.00	1.00	0.1122	0.31615
Q3	303	0.00	1.00	0.1584	0.36573
Q4	303	0.00	1.00	0.0165	0.12761
Q5	261	0.00	1.00	0.8927	0.31006
Q6	260	0.00	1.00	0.8923	0.31059
Q7	258	0.00	1.00	0.2442	0.43044
Q8	303	0.00	1.00	0.0759	0.26529
Q9/1	232	0.00	1.00	0.6983	0.46000
Q9/2	229	0.00	1.00	0.2052	0.40476
Q10	303	0.00	1.00	0.1089	0.31204
Q11	219	0.00	1.00	0.3379	0.47408
Q12	215	0.00	1.00	0.2465	0.43199
Q13	303	0.00	1.00	0.0363	0.18735
Q14	210	0.00	1.00	0.4810	0.50083
Q15	303	0.00	1.00	0.1947	0.39664
Q16	207	0.00	1.00	0.3237	0.46901
Q17	205	0.00	1.00	0.5317	0.50022
Q19	210	0.00	1.00	0.7000	0.45935

*Note:* Item difficulty was expressed as the mean proportion of correct responses (*p*‐value), ranging from 0 (very difficult) to 1 (very easy).

## 4 Discussion

A total of 303 clinicians completed the questionnaire, mostly ICU nurses with > 5 years of experience, and 7% answered all questions correctly. Zeroing procedures were understood by 11%–16%, while knowledge of correct transducer placement was limited (2%–21%). Although 77% recognised the effect of transducer height changes on displayed pressure, half answered scenario‐based questions correctly. Identification of the phlebostatic axis and reference landmarks was low (4%–33%), though experienced staff performed better. Practical handling varied, with 48% correctly inflating pressure bags and most avoiding heparin in flush solutions. Larger hospitals showed higher accuracy.

Although the evidence regarding the degree of numerical precision required for haemodynamic decision‐making is limited [17] accurate monitoring is generally considered important. While absolute accuracy thresholds remain uncertain, procedural correctness—particularly with respect to levelling and zeroing—helps minimise avoidable systematic error in the measured values.

### 4.1 Competencies in Haemodynamic Monitoring

The analysis of research‐ and teaching‐related competencies in haemodynamics showed that only a very small proportion of *n* = 4 (1%) respondents had experience in study design. The vast majority of *n* = 293 (97%) had no such experience while a few of *n* = 3 (1%) were unable to provide a clear answer. A higher proportion of *n* = 35 (12%) reported having attended training in haemodynamic monitoring within intensive care. Most with *n* = 266 (88%) had not participated in or taught such courses. Overall, these findings indicate limited scientific experience among respondents, although a relevant minority of *n* = 35 (12%) demonstrated expertise in haemodynamic monitoring. These findings correspond with those of Xiao et al. from China [21], who highlighted that inadequate training of ICU nurses in managing arterial catheters—particularly in maintaining arterial line patency—was reflected in their results.

### 4.2 Zeroing—Levelling

Only *n* = 34 (11%) of participants correctly identified the purpose of zeroing and *n* = 48 (16%) the required conditions. These low values are consistent with the findings of Schortgen and Le Bec from France [12], who also reported frequent confusion between zeroing and levelling, as well as uncertainty in defining the phlebostatic reference point. Several factors may explain these deficits. First, during informal on‐the‐job training, basic principles are often taught in a highly practice‐oriented manner, which may reinforce local, non‐evidence‐based routines [12]. Second, training practices differ across professions and institutions, hindering the establishment of standardised approaches [12]. Third, the multiple‐response format used in this survey was methodologically demanding. Sedlmeier et al. from Germany note that such formats place higher demands on working memory and increase the likelihood of errors, particularly when knowledge is incomplete [20]. Combined with the complexity of the topic—namely the need to clearly distinguish between zeroing and levelling—this may have further reduced the proportion of correct responses. Clinically, these gaps in competence are significant, as incorrect zeroing can cause systematic measurement errors, potentially compromising haemodynamic management and patient safety. Given the narrow target ranges for mean arterial pressure in modern intensive care, a standardised and accurately performed zeroing procedure appears essential. More research is needed for identifying and adjusting the most efficient training modules in this topic.

### 4.3 Transducer Height

Different anatomical landmarks have historically been suggested for transducer placement [22]. In practice, the right atrium has become the standard reference, corresponding in the supine patient to the fourth intercostal space at the mid‐axillary line (phlebostatic axis) to avoid hydrostatic errors [23]. Levelling compensates for hydrostatic pressure differences [24]. In this survey, only *n* = 131 (36.1%) identified the correct reference. Ideally, the catheter tip should also lie at this level, feasible only with certain sites, such as the radial artery. Ortega et al. from the United States [25] recommend alignment with the structure of greatest interest. Since the first central venous pressure measurements [26], the exact reference height has remained debated. While less relevant for arterial pressures, it is critical for low‐pressure and intracranial measurements [27, 28, 29]. For example, if MAP falls below cerebral autoregulation limits (50–150 mmHg), cerebral oxygenation drops significantly [30]. More than half of respondents with *n* = 165 (55%) fixed the transducer on a mounting plate, requiring readjustment with patient posture. Only *n* = 5 (2%) answered the question on correct mounting height, while *n* = 233 (77%) recognised the effect of height changes. Experienced staff answered more often correctly (94% vs. 76%, *p* < 0.001). These findings indicate basic understanding of hydrostatic principles but major gaps in reference height knowledge, underscoring the need for standardised training.

### 4.4 Practical Handling

Pressure transducers convert oscillatory signals into electrical signals displayed on the monitor [25, 31]. In this study, only *n* = 33 (11%) identified correct measures for a damped arterial waveform. Correct interpretation of waveform characteristics and elimination of under‐ or overdamping is essential [24]. The square‐wave flush test is recommended to detect such conditions [32]. Zeroing does not need to be repeated after every position change, except during air transport, although guidelines sometimes imply otherwise [33]. Almost half of respondents with *n* = 147 (48%) reported correct pressurisation of the flush system to 300 mmHg, ensuring continuous flow of 3–6 mL/h to prevent thrombosis. Most participants with *n* = 188 (65%) did not add heparin, likely to avoid heparin‐induced thrombocytopenia, leading to variable local practices. Adherence to institutional SOPs is therefore essential. The results indicate limited technical understanding of invasive pressure monitoring. More experienced clinicians and those in tertiary hospitals answered significantly more often correctly. This may reflect advanced training in intensive or anaesthesia nursing, which is recommended for at least 50% of staff in critical care [34]. Current medical curricula and specialist training provide only general coverage of pressure and haemodynamic, without standardised, detailed teaching. The German guideline for assessing haemodynamic stresses the importance of critical engagement with haemodynamic monitoring to prevent errors and misinterpretation [35]. Further research is needed to establish a robust scientific foundation.

Keog demonstrates that arterial catheters (ACs) are used very frequently in Australian and New Zealand ICUs, with a point prevalence of 68.2%, which is significantly higher compared to the USA (38%) and Europe (44%). The radial artery was the most common insertion site (87.3%), and 65.7% of ACs were connected to open transducer systems, despite evidence that closed systems may reduce infection risk and blood loss. Although 89.8% of ICUs have policies recommending ultrasound‐guided insertion, there is a lack of standardised practices, indicating room for improvement in training and implementation [36].

## 5 Limitations

This study has several limitations. First, the questionnaire was distributed via the authors' private and professional networks, which may have introduced selection bias. Second, the majority of participants were nursing professionals, limiting the implementation of the interprofessional approach and restricting direct comparisons between professional groups. This may reflect the clinical practice setting, as nurses are often primarily responsible for the management of arterial catheters. Third, some limitations may arise from the survey design itself. In particular, items with multiple‐response options yielded lower response accuracy, possibly reflecting a higher level of difficulty for these questions. Q4 (transducer level) might be debatable in neurosurgical patients, leading to different answers by clinicians working in the discipline, but the likely effect on main results appears to be negligible. Because some items addressed local clinical routines rather than established best practices, they reflected different underlying constructs.

The questionnaire used was not formally validated. The absence of comprehensive psychometric testing limits the interpretability of the results. Although initial steps towards assessing content and face validity were taken, full instrument validation is still lacking and should be addressed in future research. In addition, the questionnaire primarily assessed established factual knowledge and only addressed broader theoretical constructs to a limited extent, which would require more extensive psychometric validation. Accordingly, the instrument should be considered an exploratory tool rather than a fully validated measure.

Due to small sample sizes within the individual areas, subgroup analyses by clinical setting (ICU/OR/ER) were also not feasible.

## 6 Implications for Practice

Critical knowledge deficits in haemodynamic monitoring may be present among ICU clinicians, particularly regarding zeroing, transducer levelling, waveform interpretation and the phlebostatic axis. These gaps pose potential risks to patient safety and underline the need of training across professional groups. The lack of standardised nursing guidelines for invasive arterial blood pressure monitoring leads to unclear hospital procedures and threatens the quality of care [37]. The results may possibly reflect this issue. Advanced education programmes and adherence to evidence‐based guidelines should be prioritised to ensure consistent practice. Institutions should implement competency‐based teaching, simulation‐based learning and regular assessments to consolidate theoretical knowledge and practical skills. Standardisation of protocols, combined with ongoing professional development, can reduce heterogeneity and improve the safety and reliability of invasive blood pressure monitoring.

## 7 Conclusions

This survey indicates notable gaps in knowledge related to haemodynamic monitoring among ICU clinicians, particularly regarding zeroing, transducer levelling, waveform interpretation and the phlebostatic axis. While fundamental understanding of hydrostatic principles was present, precise application of reference levels and procedures remained limited. Approaches to practical handling, such as pressure bag use and heparin administration, also varied. More experienced professionals and those in tertiary centres demonstrated higher accuracy, reflecting the value of training and institutional standards. These results highlight the importance of strengthening structured education, advanced training programmes and clear evidence‐based guidelines to further support safe patient care.

## Funding

The authors have nothing to report.

## Disclosure

The authors have nothing to report.

## Ethics Statement

The authors have nothing to report.

## Consent

The authors have nothing to report.

## Conflicts of Interest

The authors declare no conflicts of interest.

## Supporting information


**Data S1:** Supporting Information.

## Data Availability

The data that support the findings of this study are available from the corresponding author upon reasonable request.
