# Supplementary material for: INPRESS: INvasive PRESSure Monitoring Knowledge and Practice Survey
Source: Nurs Crit Care. 2026 Mar 12;31(2):e70443. doi: 10.1111/nicc.70443 (PMC12982938; doi:10.1111/nicc.70443)
Supplement: Supplementary file 1 — Data S1: Supporting Information. [file NICC-31-0-s001.docx]

**Supplement: INPRESS: INvasive PRESSure Monitoring Knowledge Survey**

**Coolens O, Dubb R, Kaltwasser A, Lorenzen U, Nydahl P.**

**Nursing in Critical Care, 2026**

This is the English version of the questionnaire.

The original survey has been developed by Schortgen and Le Bec 2008 (https://pubmed.ncbi.nlm.nih.gov/39932728/).

The online version in German language includes additional information for participants, available here https://de.surveymonkey.com/r/INPRESS2025

**1. Participant Characteristics**

1. In which setting do you work?

- IMC
- Anesthesia
- ICU
- Emergency Department

1. Which professional group do you belong to?

- Physician
- Registered Nurse
- Advanced Practice Nurse
- Other

1. How many years of work experience do you have (excluding training)?

- <6 months
- 6 months to 2 years
- More than 2 to 5 years
- 5 years

1. In which type of institution do you work?

- Primary care hospital
- Secondary care hospital
- Tertiary care hospital
- Quaternary (maximum care) hospital
- Other

1. Have you ever participated in the design of studies on hemodynamics?

- Yes
- No

1. Have you ever taught a course on hemodynamics in the ICU?

- Yes
- No

**2. Zeroing Procedure**

What is the purpose of zeroing? (Multiple answers possible)

1. Record the minimum blood pressure of the cardiovascular system
2. Record the atmospheric pressure
3. Calibrate the arterial pressure system
4. Ensure the curve drops to zero during calibration
5. Ensure the transducer is positioned at the correct height
6. I don’t know

**Correct answers:** 2, 3, 4

**3. Requirement for Zeroing**

Zeroing must be performed … (Multiple answers possible)

1. When the transducer is at the zero reference level
2. Whenever the patient has been repositioned
3. When the cable between the transducer and the monitor has been connected
4. When the arterial pressure waveform is dampened
5. Whenever the height of the transducer has changed
6. I don’t know

**Correct answer:** 3

**4. Transducer Level**

The height above the floor at which the transducer is mounted … (Multiple answers possible)

1. Corresponds to the zero reference level
2. Corresponds to the zero reference pressure
3. Must match the height of the artery whose blood pressure is being measured
4. Significantly influences the arterial pressure displayed on the monitor
5. Is determined by the height of the stopcock used for zeroing
6. I don’t know

**Correct answers:** 1, 3, 4, 5

**5. Transducer Level Too High**

If the transducer is placed too far **above** the zero reference level, the displayed arterial pressure will be …

1. Too low
2. Too high
3. Unaffected
4. I don’t know

**Correct answer:** 1

**6. Transducer Level Too Low**

If the transducer is placed too far **below** the zero reference level, the displayed pressure will be …

1. Too low
2. Too high
3. Unaffected
4. I don’t know

**Correct answer:** 2

**7. Transducer Plane**

Where do you place the transducer connected to the radial arterial catheter?

-
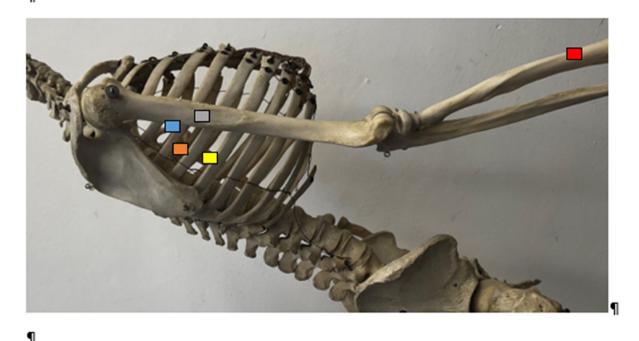
Blue
- Yellow
- Orange
- Gray
- Red
- I don’t know

**Correct answer:** Gray

**8. Correct Transducer Level**

In which figure(s) is the transducer (shown in white) placed at the correct reference level? (Multiple answers possible)


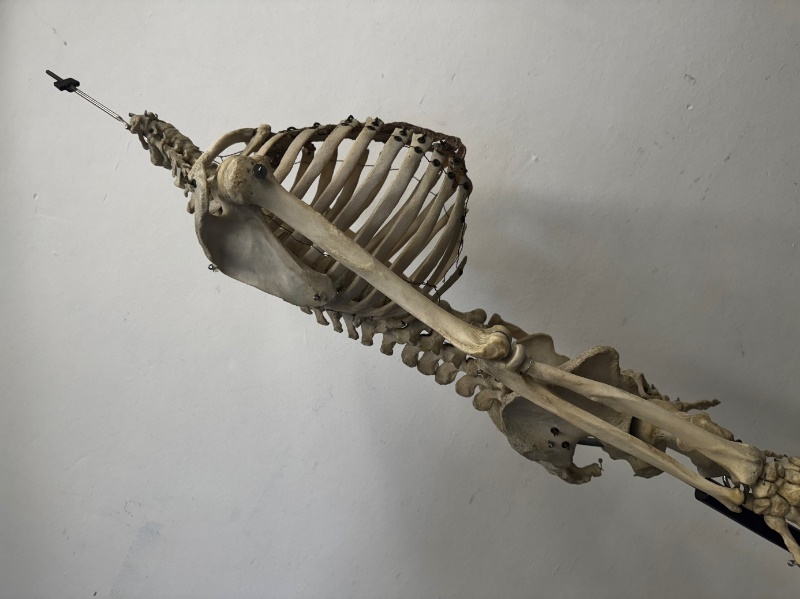

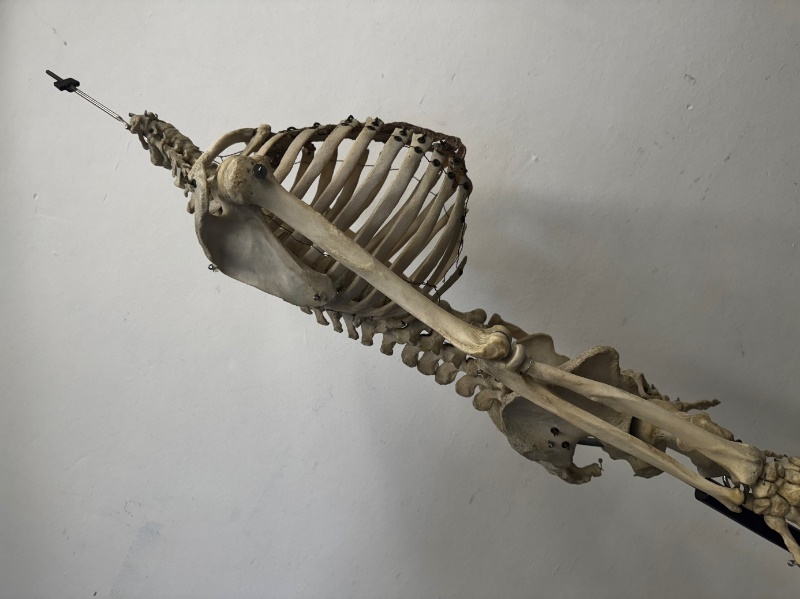


**2**

**1**


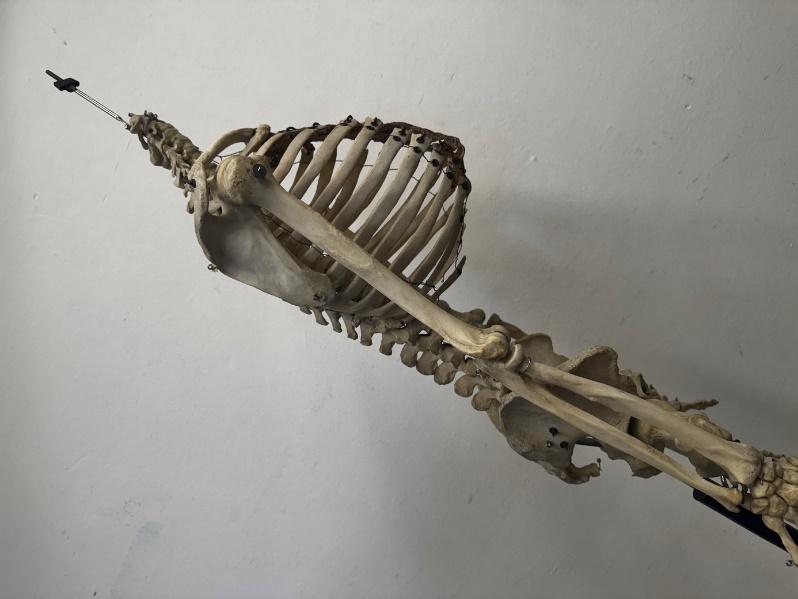

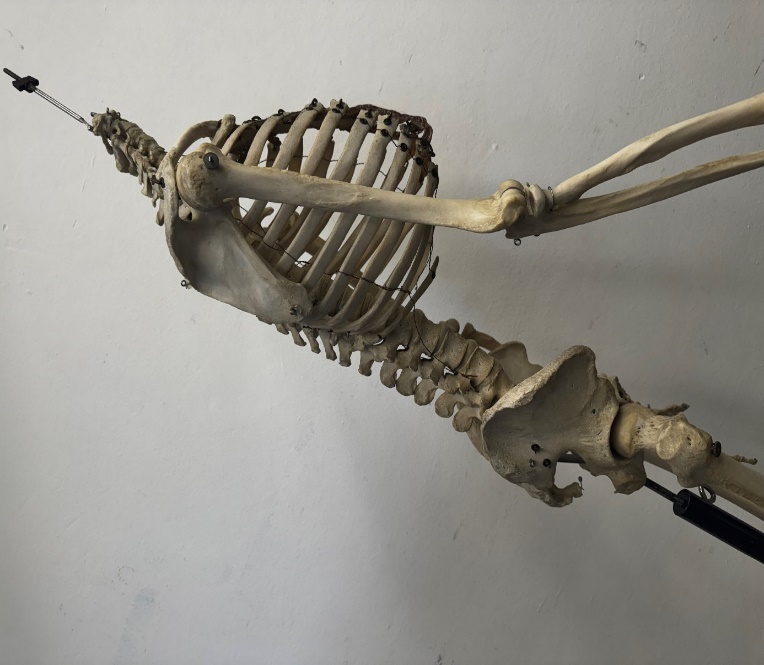


**4**

**3**


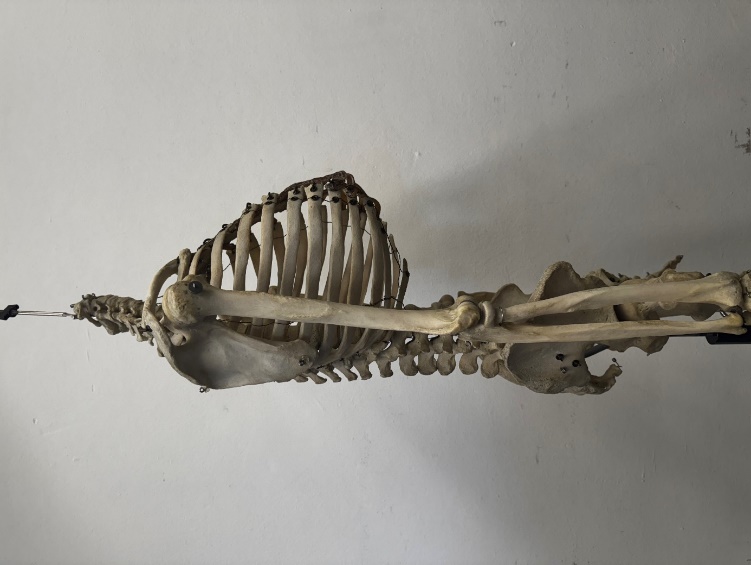

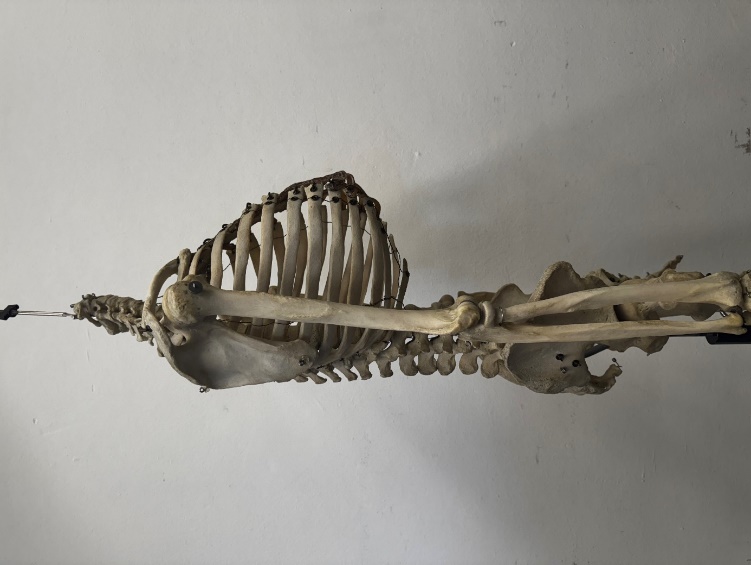


**6**

**5**

- 1
- 2
- 3
- 4
- 5
- 6
- I don’t know

**Correct answers:** 2, 4, 5

**9. Incorrect Transducer Level: Magnitude of Error**

The current mean arterial pressure (MAP) is 60 mmHg.
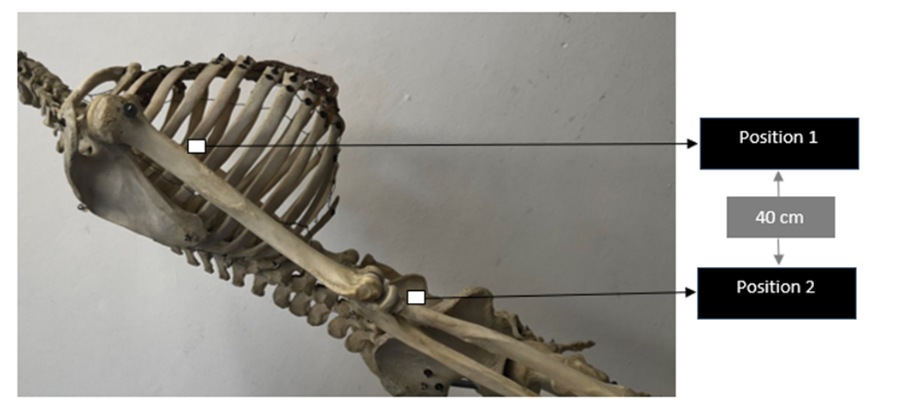


- If the transducer is at position 1, what MAP is displayed?
  - 60 mmHg
  - 70 mmHg
  - 80 mmHg
  - 90 mmHg
  - I don’t know
- If the transducer is at position 2, what MAP is displayed?
  - 60 mmHg
  - 70 mmHg
  - 80 mmHg
  - 90 mmHg
  - I don’t know

**Correct answers:** 60 mmHg (position 1) and 90 mmHg (position 2)

**10. Arterial Pressure Waveform**

You see the following arterial pressure waveform on the monitor: 95/35 (60) mmHg, HR 129 bpm.
What is your assessment and what actions would you take? (Multiple answers possible)


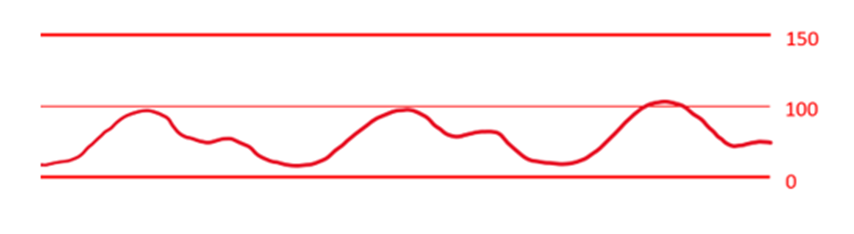


1. Waveform normal: no further action
2. Adjust the scale
3. Flush the arterial catheter
4. Check for a kink in the arterial catheter
5. Zeroing
6. Initiate further measures (e.g., echocardiography or call for help)

**Correct answers:** 2, 3, 4

**11. Displayed Invasive Arterial Pressures**

The invasive blood pressure measurements presented on the monitor reflect …

1. Zero pressure
2. The diastolic arterial pressure
3. The pressure in the atrium
4. Atmospheric pressure
5. I don’t know

**Correct answer:** 4

**12. Fluid Column and Displayed Pressure**

Using a fluid-filled system prevents a water column effect on blood pressure.

- True
- False
- I don’t know

**Correct answer:** False

**13. Phlebostatic Axis: Definition**

The phlebostatic axis … (Multiple answers possible)

1. Is the site where cardiovascular pressure is lowest
2. Corresponds to the right atrium
3. Corresponds to the superior vena cava
4. Determines the zero reference point for arterial pressure measurement
5. Determines the zero reference point for right atrial pressure measurement
6. I don’t know

**Correct answers:** 2, 4, 5

**14. Anatomical Landmark for Phlebostatic Axis**

Which anatomical landmark identifies the phlebostatic axis in a supine patient?

1. Mid-axillary line
2. Level of the nipple
3. A line 5 cm below the manubrium
4. A line 10 cm above the patient’s bed surface
5. Other
6. I don’t know

**Correct answer:** 1

**15. Validity of Phlebostatic Axis in Different Positions**

In which body position(s) can the phlebostatic axis be accurately located using its external landmark? (Multiple answers possible)

1. Supine
2. Semi-recumbent 30°
3. Prone
4. Sitting
5. Lateral
6. Trendelenburg

**Correct answer:** 1

**16. Transducer Level for Cerebral Perfusion Pressure**

To measure cerebral perfusion pressure (MAP – ICP), place the arterial transducer at the level of …

1. Phlebostatic axis
2. Arterial catheter insertion site
3. Tragus
4. It does not matter
5. I don’t know

**Correct answer:** 3

**17. Transducer Position and Aortic Root Pressure**

If the transducer is placed at the level of the right atrium, the displayed arterial pressures approximately reflect the pressure at the aortic root.

- True
- False
- I don’t know

**Correct answer:** True

**18. Transducer Placement in Practice**

Where do you most commonly place the transducer in your practice?

- On the patient’s arm
- On a mounting plate
- On the patient’s body near the arterial catheter insertion site
- On the thorax
- I don’t know

**19. Inflating the Pressure Bag**

To what pressure do you inflate the pressure bag in your practice (mmHg)?

- 100 mmHg
- 200 mmHg
- 300 mmHg
- Depends on the patient’s blood pressure
- I don’t know

**Correct answer:** 300 mmHg

**20. Heparin in the Flush Bag**

Do you add heparin to the pressure bag of the arterial system in your setting?

- Always
- Sometimes
- Never
- I don’t know
